# Ultrasonic Deposition of Cellulose Nanocrystals on Substrates for Enhanced Eradication Activity on Multidrug-Resistant Pathogens

**DOI:** 10.3390/polym17020154

**Published:** 2025-01-09

**Authors:** Lama Jabreen, Moorthy Maruthapandi, Arulappan Durairaj, John H. T. Luong, Aharon Gedanken

**Affiliations:** 1 Department of Chemistry, Bar-Ilan Institute for Nanotechnology and Advanced Materials, Bar-Ilan University, Ramat-Gan 52900, Israel; lamabiu9@gmail.com (L.J.); chemdraj@gmail.com (A.D.); 2 Department of Chemistry, Ben Gurion University of the Negev, Beer Sheva 8410501, Israel; lewismartin.jesus@gmail.com; 3 School of Chemistry, University College Cork, T12 YN60 Cork, Ireland; luongprof@gmail.com

**Keywords:** cellulose nanocrystals, ultrasonication, surface hydrophobicity, biofilm bacterial proliferation, multidrug-resistant pathogens

## Abstract

Amidst the pervasive threat of bacterial afflictions, the imperative for advanced antibiofilm surfaces with robust antimicrobial efficacy looms large. This study unveils a sophisticated ultrasonic synthesis method for cellulose nanocrystals (CNCs, 10–20 nm in diameter and 300–900 nm in length) and their subsequent application as coatings on flexible substrates, namely cotton (CC-1) and membrane (CM-1). The cellulose nanocrystals showed excellent water repellency with a water contact angle as high as 148° on the membrane. Noteworthy attributes of CNC-coated substrates include augmented reactive oxygen species (ROS) generation, heightened surface hydrophobicity, and comprehensive suppression of both drug-sensitive (*MDR E. coli* and *MRSA*) and susceptible (*E. coli* and *S. aureus*) planktonic and biofilm bacterial proliferation. In contrast, the uncoated substrates display 100% bacterial growth for the above bacteria. Empirical data corroborate the pronounced biofilm mass reduction capabilities of CNC-coated substrates across all tested bacterial strains. Elucidation of underlying mechanisms implicates ROS generation and electrostatic repulsion between CNCs and bacterial membranes in the disruption of mature biofilms. Hydroxyl radicals, superoxide, and hydrogen peroxide possess formidable reactivity, capable of disrupting essential biomolecules such as DNA, proteins, and lipids. The engineered CNC-coated substrates platform evinces considerable promise in the realm of infectious disease management, offering a cogent blueprint for the development of novel antimicrobial matrices adept at combating bacterial infections with efficacy and precision.

## 1. Introduction

Nanomaterials with well-characterized structures and functionalities have been advocated for diverse applications in various fields such as medicine, electronics, biomaterials, and energy production [1,2]. As the most abundantly available and renewable natural polymer, cellulose is considered an alternative resource of carbon compounds. Cellulose molecules consist of β-1,4 linked glucopyranose units with hydrogen bonds with two distinct domains: amorphous and crystalline. Based on its size and form, the cellulose was categorized into macroscopic fibers, microfibrillated cellulose, cellulose nanocrystals (CNC), and cellulose nanofibrils [3,4]. Cellulose nanocrystals are an emerging renewable nanomaterial isolated by a selective acid hydrolysis of cellulose fibrils or under treatment with persulfates [3]. Modified CNCs with significantly improved physical, chemical, biological, as well as electronic properties, can be developed [5]. To date, modified CNCs are potential candidates for many different applications, such as in personal care, chemicals, foods, and pharmaceuticals. CNCs are rigid rod-like crystals with diameters in the range of 10–20 nm and lengths of a few hundred nanometers [6].

Nanoparticles can be deposited on various substrates using ultrasound-assisted sonochemical deposition, resulting in a uniform distribution and strong attachment of nanoparticles on the substrates [7]. This simple approach has proven to be an outstanding technique for coating nanoparticles on ceramic, cotton, polymeric, metallic, glass, textiles, and paper [8,9,10,11]. Among these materials, fabrics are expected to have several exciting properties for new multifunctional applications including antibacterial fabrics, waterproofing, conductive fabrics, self-cleaning fabrics, flame retardancy, and super-hydrophobic textiles [12,13]. Developing a new class of multifunctional textiles is essential due to the pristine fabrics’ poor activity, toxicity, and ability to induce skin disease. Further research development on multifunctional fabrics is required to solve the hydrophobic issue and provide a favorable environment for a microorganism’s growth [14]. A variety of nanomaterials including graphene oxide, metal oxide, and metal compounds have been incorporated/functionalized on the fabric surface via sol–gel-based coating, electrodeposition, and layer-by-layer deposition techniques [15]. Among these materials, surface-functionalized cellulose nanocrystals are inexpensive and have unique properties such as low weight, high aspect ratio, high mechanical strength, chemical modification, and thermal properties [3,16,17,18]. The presence of surface hydroxyl groups on the CNCs may permit further modification to alter its hydrophilicity, provide a stabilization matrix for anchoring metallic nanoparticles, or prepare the biomaterial for targeted applications [19]. One of the most challenging issues in clinical therapy is drug-resistant bacterial infection wounds. Hemostasis, anti-inflammation, proliferation, and re-epithelialization are several overlapping processes that arise during the healing of skin wounds [20]. Shortening the hemostasis period can encourage the wound to progress into the healing stage sooner since the wound starts to heal once hemostasis finishes [21]. Endogenous antibacterial substances, including metal nanoparticles, organic antibacterial substances, quaternary ammonium salts, and high-molecular-weight polymers with amino groups have been thoroughly studied in recent years for the treatment of bacterially infected wounds, and offer long-lasting antibacterial properties [22,23,24,25,26]. The disadvantage is that it is insufficient to eliminate biofilms and suppress resistant microorganisms.

This study describes the use of ultrasonication to prepare CNC nanoparticles, which are coated onto cotton and membrane, two widely used substrates. The main strategy is to explore some unique characteristics of CNC by introducing an ultrasonic method, hence providing cost-effective and promising bionanomaterials as an alternative to metal nanoparticles. The CNC/Membrane and CNC/Cotton are analyzed using various spectroscopical analyses such as XRD, HRTEM, ESEM, and XPS. The coated substrates are used against *Escherichia coli* (*E. coli*), *Staphylococcus aureus* (*S. aureus*), multidrug-resistant *E. coli* (MDR *E. coli*), and methicillin-resistant *S. aureus* (MRSA), considering a broad spectrum in the anti-microbial activity of CNCs. The coated materials’ antibiofilm activities on surface charge and hydrophobicity will be assessed using the appropriate spectroscopic and microscopic techniques.

## 2. Experimental

### 2.1. Materials and Chemicals

Microcrystalline cellulose (MCC- particle size between 50 and 60 µm), Nylon 66 (polyamide), and sulfuric acid (H_2_SO_4_, 98%) were purchased from Sigma Aldrich, Jerusalem Israel. The commercial cellulose nanocrystals (CNCs) were purchased from Nanografi Nano Technology, Turkey (https://nanografi.com/). These nanocrystals are 10–20 nm in diameter and 300–900 nm in length. Deionized water was used in all the experiments. The cotton was purchased from a textile company, Klopman Int. Frosinone, Italy (https://www.klopman.com/). All the acquired chemicals and solvents were utilized without any purification.

### 2.2. Preparation of CNCs

MCC was subjected to acid hydrolysis treatment using sulfuric acid under the influence of ultrasonic radiation. The hydrolysis was carried out by introducing 0.5 g of MCC into 10% (*w*/*w*) of sulfuric acid to form a suspension. This suspension was held at 60 °C under sonication for 60 min (Ultrasonic Disruptor, 20 kHz, 675 W, 35% amplitude, power density of 0.37 W·cm^−3^) to allow hydrolysis to take place. Immediately, following the hydrolysis, the suspension was transferred into centrifuge bottles, centrifuged at 12,000 rpm for 10 min, and decanted to separate the crystals. The residual materials were washed with distilled water and the mixture was centrifuged again to remove traces of sulfate salts [27].

### 2.3. Coating Method

**Scheme 1:**
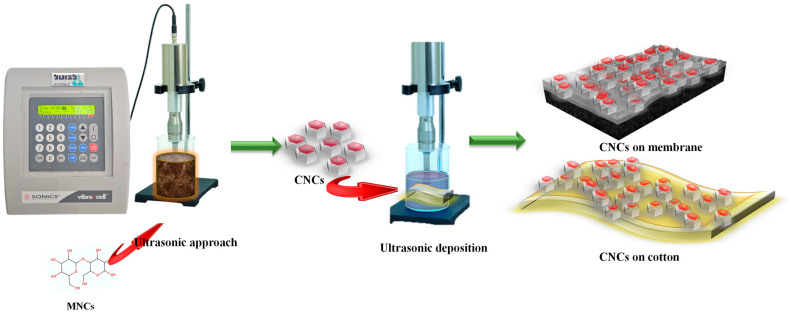
The Preparation of CNC and ultrasonic surface coating process.

The CNC-coated membrane and cotton were prepared by ultrasonic method. The media membrane 2 cm^2^ was dipped in 0.1% (wt.) of CNCs, and undergoing sonication for 30 min, 30% amplitude allowed the cellulose NPs to adhere to the whole surface and create a uniform film [28,29]. In the case of the CNC-coated cotton sample, 1% (wt.) of CNCs were used to deposit on the cotton surface (2 cm^2^). The CNC-coated samples were immersed in water and ethanol to remove the unbonded CNC particles from the substrate surface and dried in air overnight at 60 °C. The schematic illustration of CNC sysnthesis and coating process shown in Scheme 1.

### 2.4. Characterization Methods

The surface morphology and the size of the CNC coating on the surface of the fabrics were characterized by high-resolution scanning electron microscopy (HR-SEM) using a Magellan 400 L (FEI-Thermo fisher scientific), USA device at 5 kV. The crystalline nature of the microcrystalline cellulose and the synthesized cellulose nanocrystals were assessed using an X-ray diffraction (XRD) technique measured using a Bruker Inc. Germany AXS D8 Advance diffractometer. ROS generation was measured using electron paramagnetic resonance (EPR) spectroscopy. The particle size and morphology of CNCs were probed with a transmission electron microscope with the model number TEM-JEOL-2100 (Peabody, MA, USA). The CNC powder was deposited on the copper grid and dried overnight at 60 °C. For contact angle measurement at 25 °C, the coated substrate was placed in a contact angle goniometer, and water droplets were deposited on each surface.

## 3. Results and Discussion

This study sought to prepare superhydrophobic and antibacterial textiles with sustainable, renewable, and non-toxic cellulose nanocrystals, which can be performed using a short one-step ultrasound-assisted procedure. The sonochemical method involves radical and/or thermal reactions, which originate from the extremely high temperatures and pressures generated in the collapse of cavitation bubbles [30]. The cellulose molecules with a very low vapor pressure reside in the surrounding surface of collapsed bubbles. CNCs are formed due to the implosive collapse of acoustic bubbles when they reach unstable sizes.

Upon the collapse of the bubble, the particles quickly move toward the center and interact together so that van der Waals or hydrogen bond forces hold them together, leading to the formation of CNCs. At the part of the coating, we have an additional occurrence: the after-effect of the bubble’s collapse. What occurs after this event is the creation of microjets in the liquid. These jets move at a very high speed (500 m/s) directed towards the solid surface. The microjets take the newly formed CNCs and throw them onto the solid substrate at this high speed so that they are embedded in the solid surface.

As shown in Scheme S1, the hydrolysis of sulfuric acid has two different paths. First, the β-1-4-glycosidic bond is broken down owing to a rapid protonation of an oxygen atom at this bond. The second path shows cyclic oxygen in a glucopyranose ring by protons from the acid. Afterward, these two different paths continue to react with water to break down cellulose, resulting in shorter cellulose fragments [30]. Simultaneously, generated SO_4_^−2^ reacts with hydroxyl groups of CNCs (Scheme S1), and this esterification reaction proceeds to yield acid half-ester and promote surface oxidation. Negatively charged CNCs-(O-SO_3_^2^) exhibit strong electric double-layer repulsion forces to prevent their self-aggregation, which is driven by hydrogen bonds [31]. Consequently, most of the amorphous regions are broken down from the cellulose long chain, resulting in shorter CNCs with high crystallinity. Some hydroxyl groups in CNCs can also be converted into sulfate groups, depending on the hydrolysis reaction time [32].

**Figure 1:**
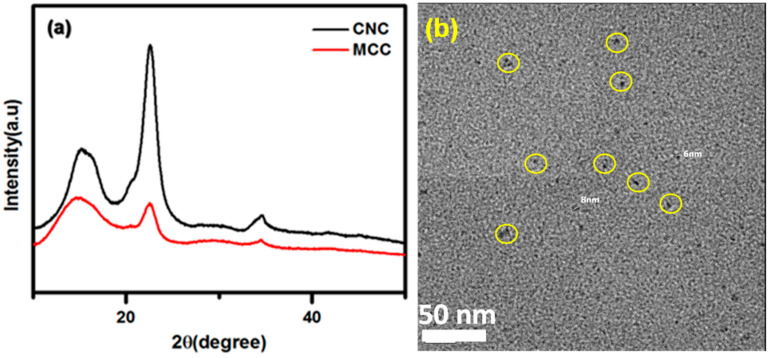
(**a**) X-ray diffraction and (**b**) TEM image of the CNCs.

To verify the validity of the prepared CNCs, the sonicated particles were characterized by TEM and XRD. X-ray diffraction (XRD) is used to study crystalline behavior and to evaluate the relation between the crystal structure and characteristics. The XRD pattern of microcrystalline cellulose and the synthesized CNCs is shown in Figure 1a. The sample exhibited peaks at 2*θ* = 34.45°, 22°, and 15°. The identical diffraction peaks indicate the success of the acid hydrolysis treatment. The XRD patterns of synthesized CNCs are confirmed by the previous literature reports [33,34].

Transmission electron microscopy (TEM) was used to examine the CNC particle size. TEM morphology image and particle distribution of CNCs derived from acid-hydrolyzed sulfuric acid-treated are shown in Figure 1 and Figure S1a. Ultrasonic dispersed CNCs exhibit particle size ranges between 5 and 10 nm due to the ultrasonic energy that disturbs the CNC surface, forming short-chain cellulose particles [35]. The TEM images in Figure S1b illustrate the commercial CNC suspension, displaying cluster nanowhiskers. Figure S1c,d shows the TEM images of microcrystalline cellulose and the SAED pattern. The MCC exhibited a cluster of cellulose fibers with 0.5–1 μm.

**Figure 2:**
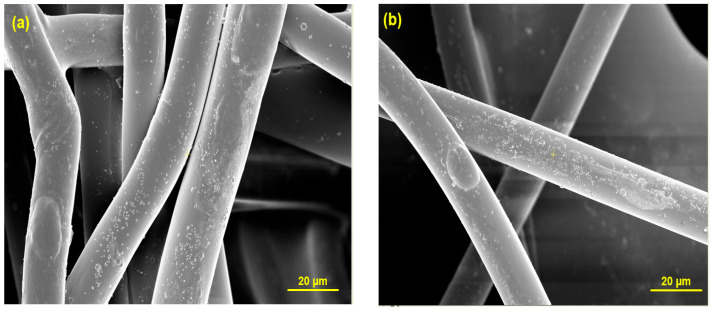

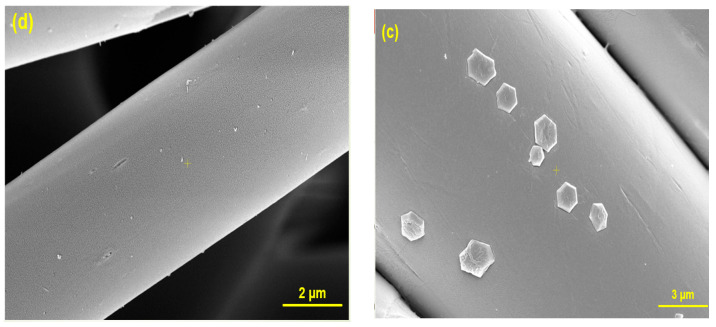
Scanning electron microscopy (SEM) images of (**a**,**b**) media membrane coated with synthesized CNCs (**c**) without coating, and (**d**) CNCs show the formation of the hexagons on the membrane with high magnification.

The surface morphology of the substrates (media membrane and cotton fabric) coated with CNCs was examined using HR-SEM after performing the sonochemical process on the cellulose solution as the coated sample and on the clean textiles as a control sample. Figure 2a,b shows the nanoparticles of the CNCs distributed on the surface with sizes of 40–70 nm. Often, the particles are aggregated on the membrane to form hexagons (Figure 2d). This morphology is compared with the control sample (Figure 2c) which shows a clean and smooth surface.

**Figure 3:**
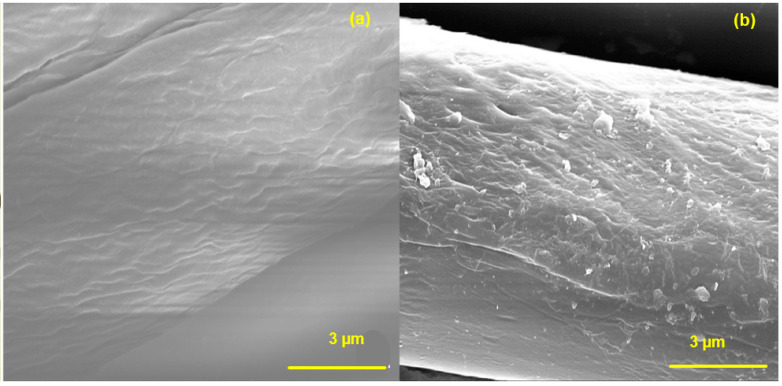
Scanning electron microscopy (SEM) images: (**a**) uncoated cotton fabric, (**b**) cotton fabric coated with CNCs.

The results in Figure 3 present the comparison between the control sample of the cotton fabric (Figure 3a) and the cotton fabric coated with CNCs (Figure 3b).

**Figure 4:**
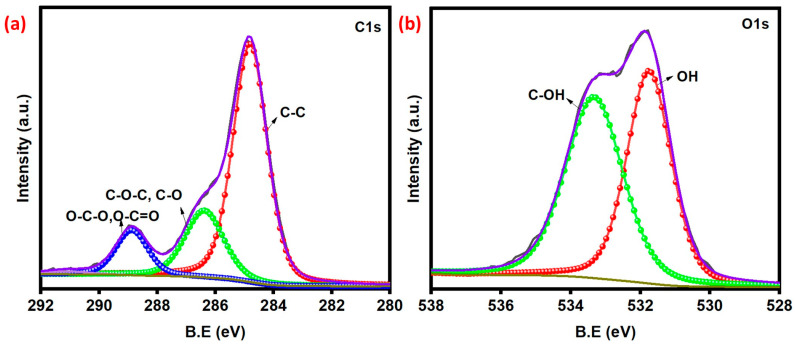
XPS spectra of Cellulose nanocrystal modified Membrane (**a**) C1s (Red: C-C, green: C-O-C, C-O and blue: O-C-O, O-C=O and (**b**) O1s (Red: OH, green: C-OH) peaks are deconvoluted from the XPS spectra. [Black-Measured; Purple: Fitted; Dark Yellow: Background].

The X-ray photoelectron spectroscopy patterns of the CNC-coated membrane are shown in Figure 4, which predominantly contains the carbon and oxygen elements. As shown in Figure 4a, the C1s spectrum shows three deconvoluted peaks at 284.8 eV, 288.3 eV, and 288.8 eV, corresponding to C-C, C-O-C/C-O, and O-C-O/O-C=O functionals groups [36]. The deconvolution of the O1s spectrum (Figure 4b) shows two peaks at 531.3 eV, and 533.3 eV have been assigned to O-H and C-O-H/C-O-C functional groups [37].

### 3.1. Contact Angle Measurement

**Figure 5:**
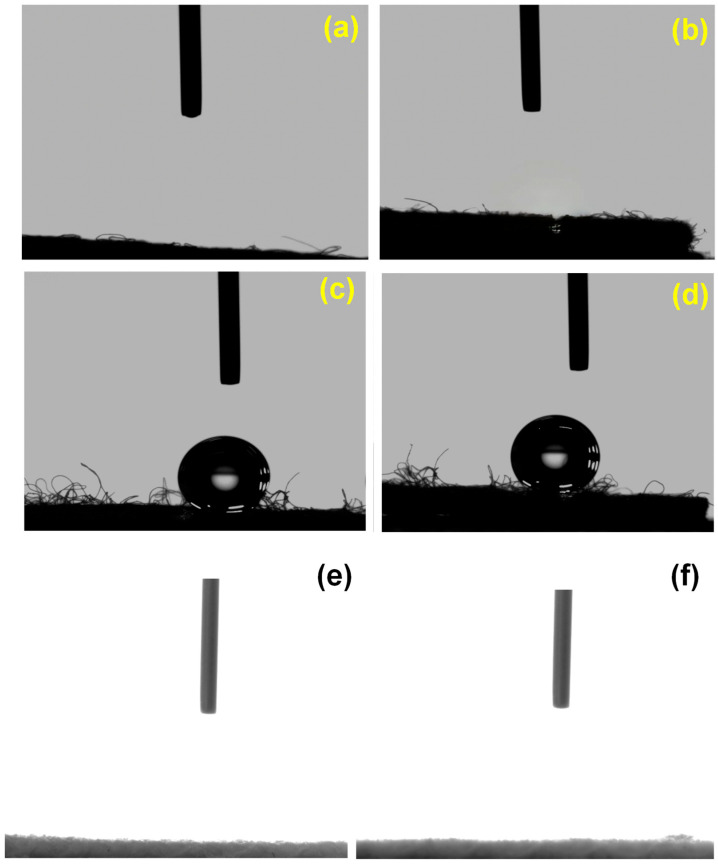
Water contact angle measurement of (**a**) uncoated media membrane, (**b**) MCC coated Media Membrane, (**c**) commercial CNCs coated membrane, (**d**) synthesized CNCs coated membrane, (**e**) uncoated cotton fabric, (**f**) CNCs coated cotton fabric.

The surface wettability of the pristine and CNC-coated fabric and membrane was assessed using water contact angle measurements. In Figure 5a, the pristine media membrane exhibited no water contact on the surface. The native cellulose (MCC) coated membrane did not show any water contact in Figure 5b. A drop of water on the commercial CNC-coated media membrane was extremely spherical with a water contact angle (WCA) of 155°, corroborating its hydrophobicity (Figure 5c). This measurement was also performed on the surface that was coated by the sonochemical synthesized CNCs, which was spherical with a water contact angle of 148° (Figure 5d). When a water drop was placed on the pristine cotton and CNC-coated cotton fabric, the water was absorbed immediately (Figure 5e,f). This difference in the hydrophobicity of the two textiles is related to the fabric/structure of the membrane, due to the porous and void nature of substrates and the hydrophobic nature of CNC particles.

### 3.2. In Vitro Antibacterial Assay

The antibacterial properties of CNC-coated substrates are widely used in a variety of applications, particularly in medical applications that come in close contact with human skin; as a result, they should be low toxicity or biocompatible [38]. Both sensitive (*E. coli* and *S. aureus*) and multi-drug resistant (MDR *E. coli*, MRSA) microorganisms were used to investigate the antibacterial properties of CNC-decorated substrates. The uncoated materials showed no reduction in the growth of drug-sensitive and -resistant *E. coli* and *S. aureus*. Bacterial membranes consist of carboxyl, amino, and phosphate groups, thus, they carry a net negative charge or the zeta potential at physiological pH. Many Gram-negative bacteria also have fimbriae, a proteinaceous appendage, which is thinner and shorter than a flagellum. These protein structures enable bacteria to circumvent electrostatic repulsion and even form strong bonds to surfaces with different negative charges. Gram-negative bacteria, lipopolysaccharide, a surface polymer, can promote bacterial attachment to negatively charged surfaces. To avoid physicochemical interactions, bacteria can alter the structure and chemical makeup of their lipopolysaccharide layer [39]. *E. coli* uses curli, which are amyloid fibers, to resist repelling forces and adhere to negatively charged particles. The directed attachment of *E. coli* to polystyrene particles with a negative charge is noteworthy. *Streptococcus mutans*, however, adheres less strongly to surfaces that are positively charged [40]. Besides the surface charge and charge density, the regulation of bacterial adhesion to surfaces also depends on other surface characteristics including roughness, stiffness, wettability, and topography [41]. Gram-negative bacteria possess acidic and basic functional groups, stemming from lipopolysaccharides (LPS) and phospholipids, versus peptidoglycan and teichoic acid for Gram-positive bacteria. Such functional groups exhibit different zeta potentials to play an important role in cellular electrostatic behavior and adhesion [42]. Gram-negative *E. coli* with an additional negatively charged LPS layer exhibits a higher zeta negative potential of over *S. aureus*.

Cellulose molecules are esterified by sulfuric acid to form the covalent coupling of sulfate groups on their surface, i.e., they are sulfated and negatively charged. Sulfated CNCs with increasing sulfate groups possess a more negative surface with low z-potential values, down to −66.1 ± 1.5 mV [43]. Therefore, one would expect a strong repulsive force between CNCs and bacterial membranes to prevent bacterial attachment on the CNC-decorated substrates, i.e., no biofilm formation as observed in this study.

**Figure 6:**
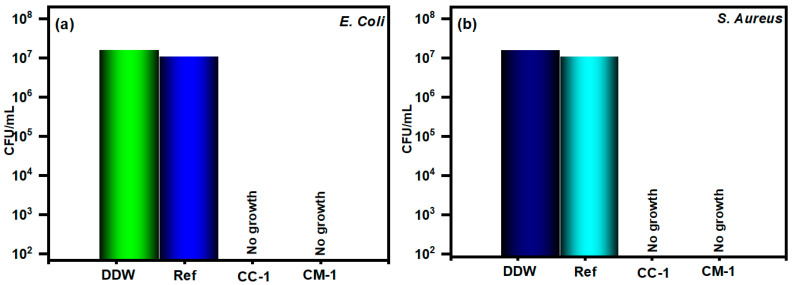

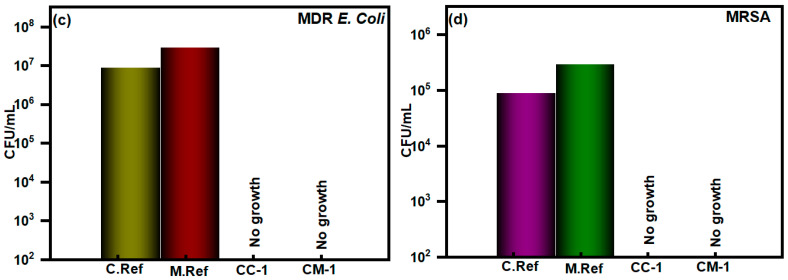
Antibacterial activity of CNCs coated substrates (**a**) Activity against *E. coli*, (**b**) *S. aureus* (**c**) MDR *E. coli*, and (**d**) MRSA.

The sonochemical and commercial CNCs were studied for their antibacterial activity against four various Gram-positive and Gram-negative bacteria. The CNCs demonstrated superiority for the eradication of Gram-positive bacteria of *S. aureus* and Methicillin-resistant *S. aureus* (MRSA) as well as toward Gram-negative bacteria *E. coli* and multidrug-resistant (MDR) *E. coli*. The results indicate that the sonochemical and commercial CNCs that were coated membranes, and the cotton fabric have a rapid response to growth inhibition on the four various bacteria that were checked (Figure 6). The CNCs coated CC-1 and CM-1 show complete growth inhibition of sensitive *E. coli* and *S. aureus* in Figure 6a,b and strong antibiotic pathogens of MDR *E. coli*, MRSA in Figure 6c,d, whereas the uncoated substrates display 100% bacterial growth. The antibacterial differences in these CNC-coated fabrics can be associated with the synthesis and loading of CNCs on the polymer fabric. Two distinct electrical potential and nanoparticle size approaches can successfully prevent bacterial growth on CNC-coated substrates.

### 3.3. Inhibition/Rupture of Biofilm Formation

**Figure 7:**
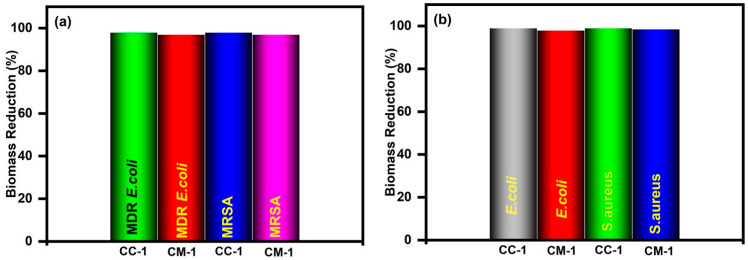
Biomass reduction in the CNCs coated substrates (**a**) MDR *E. coli* and MRSA and (**b**) *E. coli* and *S. aureus*.

The planktonic method [44] was conducted to assess the antimicrobial effect of the CNC-decorated substrates against sensitive *E. coli* and *S. aureus* pathogens and drug-resistant (MRSA, MDR *E. coli*). All the substrates exhibit remarkable antimicrobial effects against all tested pathogens as illustrated by the CFU/mL reduction and corresponding biomass reduction (Figure 6a–d). In particular, CNC-decorated substrates of CC-1 and CM-1 exhibited complete biofilm mass reduction ability for all four strains (Figure 7a,b). These results demonstrated that CNC-decorated substrates exhibit superhydrophobicity and effective antimicrobial properties. This is the first report on the complete growth inhibition against common sensitive and drug-resistant bacteria by CNC-decorated substrates [45]. Recently, there are various complex materials have been used for the microbial applications which are listed below in Table 1. The notable efficacy of the CNC-decorated materials might play an important role in further investigation against other pathogens in clinical settings. 

### 3.4. EPR Measurements

Reactive oxygen species (ROS) play a dualistic role within bacterial metabolism, arising as natural byproducts when oxygen undergoes undesired reduction states, precipitating the formation of peroxides, superoxides, and free hydroxyl radicals. These ROS, while integral to basic bacterial metabolic processes, can reach detrimental levels, inflicting cellular damage or culminating in cell death. The ingress of oxygen into bacterial cells catalyzes the production of ROS, a phenomenon notably exacerbated in aerobic conditions, where carbon nanoclusters (CNCs) can further amplify ROS concentration. This amplification of ROS concentration underpins one of the principal bactericidal mechanisms of metal nanoparticles, as expounded upon by Slavin et al. (2017) [56]. Hydroxyl radicals, along with superoxide and hydrogen peroxide, possess formidable reactivity, capable of disrupting essential biomolecules such as DNA, proteins, and lipids. Despite bacteria’s possession of protective proteins capable of detoxifying select ROS, their defense mechanisms falter against the onslaught of hydroxyl radicals, whose pervasive reactivity extends to virtually all cellular constituents.

**Figure 8:**
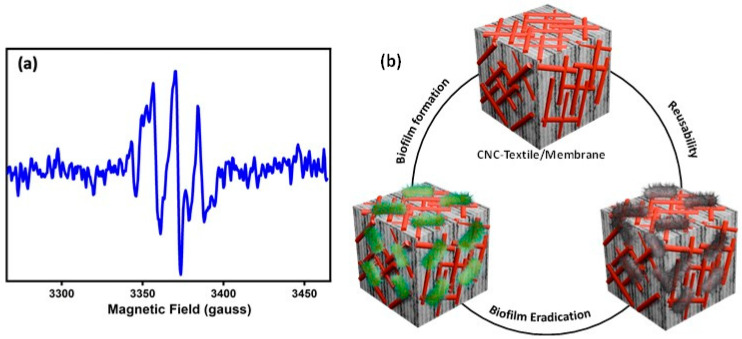
(**a**) EPR spectrum of CNCs. (**b**) Plausible biofilm eradication mechanism of CNC textile/membrane.

The intricate interplay between ROS and cellular components lies at the heart of bacterial oxidative stress. Carbon nanoclusters (CNCs) serve as potent generators of ROS, as elucidated through meticulous electron paramagnetic resonance (EPR) analyses. In these analyses, the utilization of 5-tert-butoxycarbonyl-5-methyl-1-pyrroline-N-oxide (BMPO) as a spin trap facilitates the precise quantification of ROS generation. BMPO, upon interception of hydroxyl radicals and superoxide anions, transforms BMPO-OH, manifesting as discernible signals in EPR spectra (refer to Figure 8). These free radicals wield influence over the intricate dynamics of the cell membrane, thereby modulating surface-level cell growth activities and signaling cascades. Notably, oxidative DNA damage stands as a well-recognized primary instigator of cellular demise, further underscoring the pivotal role played by ROS in bacterial pathophysiology.

In recent years, there has been a burgeoning interest in sonochemical coatings as a sophisticated method for imbuing surfaces of medical devices with biocidal properties. These coatings have been extensively explored for various applications, including the incorporation of cellulose nanocrystals (CNCs) onto textile fabrics such as membranes and cotton substrates. The notable advantage of employing sonochemical techniques lies in their capacity to firmly embed particles onto surfaces, thereby mitigating the risk of particle leaching, as elucidated by Hoo et al. [57].

**Figure 9:**
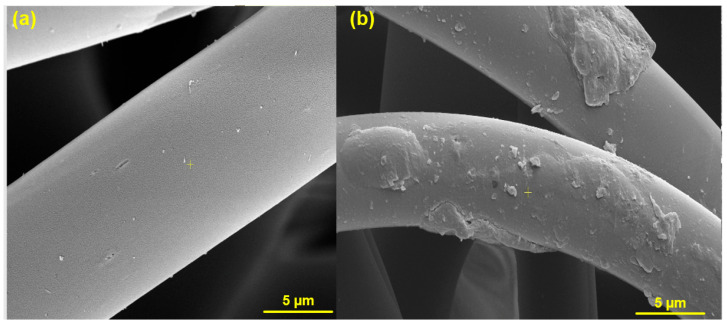
Scanning electron microscopy (SEM) images of (**a**) control sample of media membrane without coating and (**b**) membrane coated with MCC.

**Figure 10:**
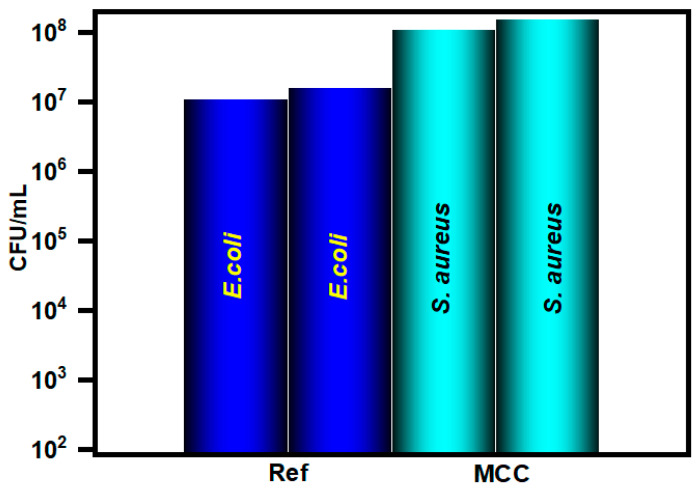
Antibacterial activity against *E. coli* and *S. aureus* on MCC-coated membrane.

The efficacy of cellulose nanocrystals as antibacterial agents stems from their ability to trigger the generation of reactive oxygen species (ROS), a phenomenon crucial for disrupting bacterial membranes. This mechanism has been corroborated through Electron Paramagnetic Resonance (EPR) tests, as evidenced by the presence of radicals, as depicted in Figure 8. Additionally, the superhydrophobic nature observed in coated fabrics can be attributed to the inward bending of hydroxyl groups within the cellulose polymer. Furthermore, the synergistic effect of CNCs is attributed to the presence of free electrons within the polymer chain, facilitating enhanced ROS production, as elucidated by You et al. (2021) [58]. In an attempt to unravel the pivotal role of nanoscale dimensions in conferring these properties through surface coatings, we conducted comparative analyses involving microcrystalline cellulose (MCC) coatings. Intriguingly, contrary to expectations, our findings reveal a lack of hydrophobicity, as evidenced by contact angle measurements depicted in Figure 5, despite proficient embedding of MCC particles within the solid surfaces, as illustrated in Figure 9. Moreover, assessments of antibacterial activity, as depicted in Figure 10, underscore a notable absence of growth inhibition against *Escherichia coli* and *Staphylococcus aureus*, further highlighting the unique efficacy of CNC-based coatings.

The cytotoxicity of CNCs deserves a brief discussion as this issue has been addressed in some pertinent reports. CNCs are expected to be biocompatible because they are prepared from cellulose, which is widely used as a thickener and filler in foods and drugs. CNCs are likely non-hazardous when ingested in small quantities from the study of their in vivo toxicity in rats [59]. However, dose-dependent toxicity has been observed for CNC concentrations of 15 and 30 μg mL^−1^, as demonstrated by the release of the cytosolic enzyme lactate dehydrogenase (LDH) [49]. As the surface charge of CNCs exhibited an important role in cellulose uptake or cytotoxicity, a simple modification of the CNC surface can affect cell penetration without cellular damage [60]. CNCs derived from different biomass sources have no significant cytotoxicity against *Spodoptera frugiperda* Sf9 insect cells and Chinese hamster lung fibroblast V79 [61]. Notably, there is a plausible correlation between the inhibitory effect and the carboxylic acid contents on carboxylated CNCs. Both oral and dermal toxicity of CNCs exhibit no adverse health effects, whereas the pulmonary and cytotoxicity yield discordant results [62].

CNCs for skin barrier protection have been attempted as a versatile foundation liquid consisting of hemp/CNCs-PLA (polylactic acid). Its plausible cytotoxicity on normal HaCaT cells evaluated by the CCK-8 assay shows no significant inhibition, even at doses of 10 mg/mL [63].

## 4. Conclusions

Nanocrystalline celluloses (CNCs) exhibiting a wide-ranging spectrum of antimicrobial activity have been synthesized through a meticulous transformation of their microsized counterparts and subsequently applied onto diverse substrates via a sonochemical methodology devoid of supplementary reagents. The prepared CNC exhibited a 6–8 nm average particle size in the TEM images. The CNCs coated membrane and cotton showed 100% growth inhibition of sensitive *E. coli* and *S. aureus.* The exceptional inhibition of bacterial growth observed on these coated substrates can be attributed to the generation of reactive oxygen species (ROS) and the intricate electrostatic interactions between CNCs and bacterial cell membranes. The involvement of CNCs in perturbing the membranes of substrates, coupled with their inherently hydrophobic nature (148°), further bolsters the inhibition of bacterial colonies. In summary, the facile, environmentally sustainable, and cost-effective approach to fabricating CNC-coated materials holds significant promise for a versatile platform in antibacterial and anti-biofilm applications, with considerable potential for clinical translation. Nevertheless, the ongoing exploration of CNC mechanisms and their assessment for biocompatibility in both in vitro and in vivo contexts remain a focal point of future investigations. The integration of CNCs into fabrics emerges as a promising avenue for the development of medical textiles aimed at shielding patients and healthcare personnel from pathogenic infections. Subsequent research endeavors will delve into unraveling the mechanisms underlying antibiofilm efficacy, alongside assessing CNCs’ capacity to disrupt mature biofilms characterized by diverse structural features.

## Figures and Tables

**Table 1:** Summarizes the antimicrobial properties of various complex materials.

Materials	Bacterial Strains	Treatments	Ref.
BME-activated Fe-doped polydiaminopyridine nanofusiform-mediated single-atom nanozyme	*E. coli*	Biofilm eradication	[46]
C_3_N_4_ nanodots supported single Cu atom nanozymes (Cu-CNNDs)	*S. aureus/E. coli*	Antibacterial activity	[47]
manganese-iron dual single-atom catalysts (Mn/Fe SACs)	*S. aureus/E. coli*	Antibacterial activity	[48]
(sp(2)c-COF-Ir-ppy(2) and sp(2)c-COF-Ru-bpy(2))	*MRSA*	Antibacterial activity and Biofilm eradication	[49]
peptide/PM/Pt microrobots	*MRSA*	Antibacterial activity	[50]
TiO_2_/CdS nanotube	*E. coli*	Biofilm eradication	[51]
Ag-doped ZnO	*MRSA* and *P. aeruginosa*	Biofilm eradication	[52]
Titanium Mesh halloysite nanotubes- HNT-Fe_3_O_4_	*S.aureus*	Biofilm eradication	[53]
CuO_2_-assisting-Zn single atom hybrid nanozymes	*S. aureus/E. coli*	Antibacterial activity	[54]
single-atom-doped porphyrin metal–organic framework (HNTM-Pt@Au)	*MRSA-infected osteomyelitis*	Antibacterial activity	[55]
CNC coated materials	*S. aureus*, *E. coli*, *MRSA* and *MDR*	Biofilm inhibition	This work

## Data Availability

The original contributions presented in this study are included in the article/Supplementary Material. Further inquiries can be directed to the corresponding author.

## Supplementary Materials

The following supporting information can be downloaded at: https://www.mdpi.com/article/10.3390/polym17020154/s1, Scheme S1. (a) Acid hydrolysis mechanism. (b) Esterification of CNC surfaces; Figure S1. (a) Particle distribution analysis of commercial cellulose nanocrystals CNCs (b) TEM image of commerical CNCs (c) TEM image of commerical MCC (d) SAED pattren of MCC.

## References

[1] Tang J., Sisler J., Grishkewich N., Tam K.C. (2017). Functionalization of Cellulose Nanocrystals for Advanced Applications. J. Colloid. Interface Sci..

[2] Wang F., Chang R., Ma R., Tian Y. (2021). Eco-Friendly and Superhydrophobic Nano-Starch Based Coatings for Self-Cleaning Application and Oil-Water Separation. Carbohydr. Polym..

[3] Durairaj A., Maruthapandi M., Saravanan A., Luong J.H.T., Gedanken A. (2022). Cellulose Nanocrystals (CNC)-Based Functional Materials for Supercapacitor Applications. Nanomaterials.

[4] Nagarajan K.J., Ramanujam N.R., Sanjay M.R., Siengchin S., Surya Rajan B., Sathick Basha K., Madhu P., Raghav G.R. (2021). A Comprehensive Review on Cellulose Nanocrystals and Cellulose Nanofibers: Pretreatment, Preparation, and Characterization. Polym. Compos..

[5] Chen Y., Zhang L., Yang Y., Pang B., Xu W., Duan G., Jiang S., Zhang K., Chen Y., Duan G. (2021). Recent Progress on Nanocellulose Aerogels: Preparation, Modification, Composite Fabrication, Applications. Adv. Mater..

[6] Cherhal F., Cousin F., Capron I. (2016). Structural Description of the Interface of Pickering Emulsions Stabilized by Cellulose Nanocrystals. Biomacromolecules.

[7] Csiszár E., Nagy S. (2017). A Comparative Study on Cellulose Nanocrystals Extracted from Bleached Cotton and Flax and Used for Casting Films with Glycerol and Sorbitol Plasticisers. Carbohydr. Polym..

[8] Durairaj A., Maruthapandi M., Luong J.H.T., Perelshtein I., Gedanken A. (2022). Enhanced UV Protection, Heavy Metal Detection, and Antibacterial Properties of Biomass-Derived Carbon Dots Coated on Protective Fabrics. ACS Appl. Bio Mater..

[9] Perelshtein I., Applerot G., Perkas N., Guibert G., Mikhailov S., Gedanken A. (2008). Sonochemical Coating of Silver Nanoparticles on Textile Fabrics (Nylon, Polyester and Cotton) and Their Antibacterial Activity. Nanotechnology.

[10] Perelshtein I., Ruderman Y., Perkas N., Beddow J., Singh G., Vinatoru M., Joyce E., Mason T.J., Blanes M., Mollá K. (2013). The Sonochemical Coating of Cotton Withstands 65 Washing Cycles at Hospital Washing Standards and Retains Its Antibacterial Properties. Cellulose.

[11] Li J., Tian X., Hua T., Fu J., Koo M., Chan W., Poon T. (2021). Chitosan Natural Polymer Material for Improving Antibacterial Properties of Textiles. ACS Appl. Bio Mater..

[12] Antinate Shilpa S., Kavitha Sri A., Jeen Robert R.B., Subbulakshmi M.S., Hikku G.S.O. (2023). A Review Focused on the Superhydrophobic Fabrics with Functional Properties. J. Appl. Polym. Sci..

[13] Zaman Khan M., Militky J., Petru M., Tomková B., Ali A., Tören E., Perveen S. (2022). Recent Advances in Superhydrophobic Surfaces for Practical Applications: A Review. Eur. Polym. J..

[14] Tariq H., Rehman A., Kishwar F., Raza Z.A. (2022). Citric Acid Cross-Linking of Chitosan Encapsulated Spearmint Oil for Antibacterial Cellulosic Fabric. Polym. Sci.-Ser. A.

[15] Malucelli G. (2016). Surface-Engineered Fire Protective Coatings for Fabrics through Sol-Gel and Layer-by-Layer Methods: An Overview. Coatings.

[16] Fontana D., Recupido F., Lama G.C., Liu J., Boggioni L., Silvano S., Lavorgna M., Verdolotti L. (2023). Effect of Different Methods to Synthesize Polyol-Grafted-Cellulose Nanocrystals as Inter-Active Filler in Bio-Based Polyurethane Foams. Polymers.

[17] Babaei-Ghazvini A., Vafakish B., Patel R., Falua K.J., Dunlop M.J., Acharya B. (2024). Cellulose Nanocrystals in the Development of Biodegradable Materials: A Review on CNC Resources, Modification, and Their Hybridization. Int. J. Biol. Macromol..

[18] Silvano S., Moimare P., Gryshchuk L., Barak-Kulbak E., Recupido F., Lama G.C., Boggioni L., Verdolotti L. (2024). Synthesis of Bio-Polyol-Functionalized Nanocrystalline Celluloses as Reactive/Reinforcing Components in Bio-Based Polyurethane Foams by Homogeneous Environment Modification. Int. J. Biol. Macromol..

[19] Wohlhauser S., Delepierre G., Labet M., Morandi G., Thielemans W., Weder C., Zoppe J.O. (2018). Grafting Polymers from Cellulose Nanocrystals: Synthesis, Properties, and Applications. Macromolecules.

[20] Aliakbar Ahovan Z., Esmaeili Z., Eftekhari B.S., Khosravimelal S., Alehosseini M., Orive G., Dolatshahi-Pirouz A., Pal Singh Chauhan N., Janmey P.A., Hashemi A. (2022). Antibacterial Smart Hydrogels: New Hope for Infectious Wound Management. Mater. Today Bio.

[21] Sorg H., Tilkorn D.J., Hager S., Hauser J., Mirastschijski U. (2017). Skin Wound Healing: An Update on the Current Knowledge and Concepts. Eur. Surg. Res..

[22] Elena P., Miri K. (2018). Formation of Contact Active Antimicrobial Surfaces by Covalent Grafting of Quaternary Ammonium Compounds. Colloids Surf. B Biointerfaces.

[23] Zhang D., Nie S., Xie M., Hu J. (2020). Antioxidant and Antibacterial Capabilities of Phenolic Compounds and Organic Acids from Camellia Oleifera Cake. Food Sci. Biotechnol..

[24] Yang W., Owczarek J.S., Fortunati E., Kozanecki M., Mazzaglia A., Balestra G.M., Kenny J.M., Torre L., Puglia D. (2016). Antioxidant and Antibacterial Lignin Nanoparticles in Polyvinyl Alcohol/Chitosan Films for Active Packaging. Ind. Crops Prod..

[25] Punjabi K., Bhatia E., Keshari R., Jadhav K., Singh S., Shastri J., Banerjee R. (2023). Biopolymer Coating Imparts Sustainable Self-Disinfecting and Antimicrobial Properties to Fabric: Translated to Protective Gears for the Pandemic and Beyond. ACS Biomater. Sci. Eng..

[26] Zein M.A., Asghar B.H., Almohyawi A.M., Alqahtani N.F., Alharbi A., Alkabli J., Elshaarawy R.F.M., Ismail L.A. (2024). Multifunctional Nanocomposites Integrated Green Synthesized Amphiphilic Chitosan/Thyme Extract/Nanosilver for Antimicrobial and Anti-Biofilm Applications. React. Funct. Polym..

[27] Jabareen L., Maruthapandi M., Saravanan A., Gedanken A. (2021). Effective Degradation of Cellulose by Microwave Irradiation in Alkaline Solution. Cellulose.

[28] Perkas N., Amirian G., Dubinsky S., Gazit S., Gedanken A. (2007). Ultrasound-Assisted Coating of Nylon 6,6 with Silver Nanoparticles and Its Antibacterial Activity. J. Appl. Polym. Sci..

[29] Maruthapandi M., Saravanan A., Manoj S., Luong J.H.T., Gedanken A. (2021). Facile Ultrasonic Preparation of a Polypyrrole Membrane as an Absorbent for Efficient Oil-Water Separation and as an Antimicrobial Agent. Ultrason. Sonochem.

[30] Tang F., Li Y., Huang J., Tang J., Chen X., Yu H.Y., Zhou Y., Tang D. (2021). An Environmentally Friendly and Economical Strategy to Cyclically Produce Cellulose Nanocrystals with High Thermal Stability and High Yield. Green. Chemistry.

[31] Lam E., Hemraz U.D. (2021). Preparation and Surface Functionalization of Carboxylated Cellulose Nanocrystals. Nanomaterials.

[32] d’Errico C., Börjesson J., Ding H., Krogh K.B.R.M., Spodsberg N., Madsen R., Monrad R.N. (2016). Improved Biomass Degradation Using Fungal Glucuronoyl—Esterases—Hydrolysis of Natural Corn Fiber Substrate. J. Biotechnol..

[33] Leung A.C.W., Hrapovic S., Lam E., Liu Y., Male K.B., Mahmoud K.A., Luong J.H.T. (2011). Characteristics and Properties of Carboxylated Cellulose Nanocrystals Prepared from a Novel One-Step Procedure. Small.

[34] Julie Chandra C.S., George N., Narayanankutty S.K. (2016). Isolation and Characterization of Cellulose Nanofibrils from Arecanut Husk Fibre. Carbohydr. Polym..

[35] Muthusamy V.P., Krishnakumar V. (2024). Ultrasonication-Induced Re-Dispersion of Freeze-Dried Cellulose Nanoparticles for Improving Strength and Durability of PAN Polymer Matrix. J. Dispers. Sci. Technol..

[36] Lei D., Tang Z., Zhao L., Wang Y., Du K. (2023). Macroporous Cellulose Microspheres Derived from Cigarette Butts Waste: Preparation, Characterization, and Application in Proteins Adsorption. Cellulose.

[37] Wang G., Zhang J., Lin S., Xiao H., Yang Q., Chen S., Yan B., Gu Y. (2020). Environmentally Friendly Nanocomposites Based on Cellulose Nanocrystals and Polydopamine for Rapid Removal of Organic Dyes in Aqueous Solution. Cellulose.

[38] Attia N.F., Zakria A.M., Nour M.A., Abd El-Ghany N.A., Elashery S.E.A. (2023). Rational Strategy for Construction of Multifunctional Coatings for Achieving High Fire Safety, Antibacterial, UV Protection and Electrical Conductivity Functions of Textile Fabrics. Mater. Today Sustain..

[39] Godoy-Gallardo M., Eckhard U., Delgado L.M., de Roo Puente Y.J.D., Hoyos-Nogués M., Gil F.J., Perez R.A. (2021). Antibacterial Approaches in Tissue Engineering Using Metal Ions and Nanoparticles: From Mechanisms to Applications. Bioact. Mater..

[40] Mulder R., Maboza E., Ahmed R. (2020). Streptococcus Mutans Growth and Resultant Material Surface Roughness on Modified Glass Ionomers. Front. Oral. Health.

[41] Villapun Puzas V.M., Carter L.N., Schröder C., Colavita P.E., Hoey D.A., Webber M.A., Addison O., Shepherd D.E.T., Attallah M.M., Grover L.M. (2022). Surface Free Energy Dominates the Biological Interactions of Postprocessed Additively Manufactured Ti-6Al-4V. ACS Biomater. Sci. Eng..

[42] Takashima S., Morisaki H. (1997). Surface Characteristics of the Microbial Cell of Pseudomonas syringae and Its Relevance to Cell Attachment. Colloids Surf. B Biointerfaces.

[43] Lin N., Dufresne A. (2014). Surface Chemistry, Morphological Analysis and Properties of Cellulose Nanocrystals with Gradiented Sulfation Degrees. Nanoscale.

[44] El-Khoury N., Bennaceur I., Verplaetse E., Aymerich S., Lereclus D., Kallassy M., Gohar M. (2021). Massive Integration of Planktonic Cells within a Developing Biofilm. Microorganisms.

[45] Peterson E., Kaur P. (2018). Antibiotic Resistance Mechanisms in Bacteria: Relationships between Resistance Determinants of Antibiotic Producers, Environmental Bacteria, and Clinical Pathogens. Front. Microbiol..

[46] Xu Q., Hua Y., Zhang Y., Lv M., Wang H., Pi Y., Xie J., Wang C., Yong Y. (2021). A Biofilm Microenvironment-Activated Single-Atom Iron Nanozyme with NIR-Controllable Nanocatalytic Activities for Synergetic Bacteria-Infected Wound Therapy. Adv. Healthc. Mater..

[47] Dai X., Liu H., Cai B., Liu Y., Song K., Chen J., Ni S.Q., Kong L., Zhan J. (2023). A Bioinspired Atomically Thin Nanodot Supported Single-Atom Nanozyme for Antibacterial Textile Coating. Small.

[48] Zhang M., Xu W., Gao Y., Zhou N., Wang W. (2023). Manganese-Iron Dual Single-Atom Catalyst with Enhanced Nanozyme Activity for Wound and Pustule Disinfection. ACS Appl. Mater. Interfaces.

[49] Sun B., Wang X., Ye Z., Zhang J., Chen X., Zhou N., Zhang M., Yao C., Wu F., Shen J. (2023). Designing Single-Atom Active Sites on Sp2-Carbon Linked Covalent Organic Frameworks to Induce Bacterial Ferroptosis-Like for Robust Anti-Infection Therapy. Adv. Sci..

[50] Milosavljevic V., Kosaristanova L., Dolezelikova K., Adam V., Pumera M., Milosavljevic V., Pumera M., Dolezelikova K., Adam V. (2022). Microrobots with Antimicrobial Peptide Nanoarchitectonics for the Eradication of Antibiotic-Resistant Biofilms. Adv. Funct. Mater..

[51] Villa K., Sopha H., Zelenka J., Motola M., Dekanovsky L., Beketova D.C., Macak J.M., Ruml T., Pumera M., Villa K. (2022). Enzyme-Photocatalyst Tandem Microrobot Powered by Urea for Escherichia Coli Biofilm Eradication. Small.

[52] Adam V., Pumera M., Ussia M., Urso M., Dolezelikova K., Michalkova H., Adam V., Pumera M., Ussia M., Urso M. (2021). Active Light-Powered Antibiofilm ZnO Micromotors with Chemically Programmable Properties. Adv. Funct. Mater..

[53] Mayorga-Martinez C.C., Zelenka J., Klima K., Kubanova M., Ruml T., Pumera M., Mayorga-Martinez C.C., Pumera M., Zelenka J., Kubanova M. (2023). Multimodal-Driven Magnetic Microrobots with Enhanced Bactericidal Activity for Biofilm Eradication and Removal from Titanium Mesh. Adv. Mater..

[54] Song J., Chen H., Lv Y., Yang W., Zhang F., Wang T., Liu D., Qu Y., Han L., Fu J. (2023). CuO_2_-Assisting-Zn Single Atom Hybrid Nanozymes for Biofilm-Infected Wound Healing. Chem. Eng. J..

[55] Yu Y., Tan L., Li Z., Liu X., Zheng Y., Feng X., Liang Y., Cui Z., Zhu S., Wu S. (2021). Single-Atom Catalysis for Efficient Sonodynamic Therapy of Methicillin-Resistant Staphylococcus Aureus-Infected Osteomyelitis. ACS Nano.

[56] Slavin Y.N., Asnis J., Häfeli U.O., Bach H. (2017). Metal Nanoparticles: Understanding the Mechanisms behind Antibacterial Activity. J. Nanobiotechnology.

[57] Hoo D.Y., Low Z.L., Low D.Y.S., Tang S.Y., Manickam S., Tan K.W., Ban Z.H. (2022). Ultrasonic cavitation: An effective cleaner and greener intensification technology in the extraction and surface modification of nanocellulose. Ultrason. Sonochemistry.

[58] You C., Ning L., Wu H., Huang C., Wang F. (2021). A Biocompatible and PH-Responsive Nanohydrogel Based on Cellulose Nanocrystal for Enhanced Toxic Reactive Oxygen Species Generation. Carbohydr. Polym..

[59] Roman M. (2015). Toxicity of Cellulose Nanocrystals: A Review. Ind. Biotechnol..

[60] Pelegrini B.L., Ré F., de Oliveira M.M., Fernandes T., de Oliveira J.H., Oliveira Junior A.G., Girotto E.M., Nakamura C.V., Sampaio A.R., Valim A. (2019). Cellulose Nanocrystals as a Sustainable Raw Material: Cytotoxicity and Applications on Healthcare Technology. Macromol. Mater. Eng..

[61] Mahmoud K.A., Mena J.A., Male K.B., Hrapovic S., Kamen A., Luong J.H.T. (2010). Effect of Surface Charge on the Cellular Uptake and Cytotoxicity of Fluorescent Labeled Cellulose Nanocrystals. ACS Appl. Mater. Interfaces.

[62] Male K.B., Leung A.C.W., Montes J., Kamen A., Luong J.H.T. (2012). Probing Inhibitory Effects of Nanocrystalline Cellulose: Inhibition versus Surface Charge. Nanoscale.

[63] Tang J., He H., Wan R., Yang Q., Luo H., Li L., Xiong L. (2021). Cellulose Nanocrystals for Skin Barrier Protection by Preparing a Versatile Foundation Liquid. ACS Omega.

