# Supplementary material for: Ultrasonic Deposition of Cellulose Nanocrystals on Substrates for Enhanced Eradication Activity on Multidrug-Resistant Pathogens
Source: Polymers (Basel). 2025 Jan 9;17(2):154. doi: 10.3390/polym17020154 (PMC11769134; doi:10.3390/polym17020154)
Supplement: Supplementary file 1 [file polymers-17-00154-s001.zip › polymers-3317511-supplementary.pdf]

Ultrasonic deposition of cellulose nanocrystals on substrates for enhanced eradication activity on multidrug-resistant pathogens

Lama Jabreen[1#], Moorthy Maruthapandi[2#], Arulappan Durairaj[1], John H.T. Luong[4], and Aharon Gedanken[1]

[1]Department of Chemistry, Bar-Ilan Institute for Nanotechnology and Advanced Materials, Bar-Ilan University, Ramat-Gan 52900, Israel

[2] Department of Chemistry, Ben Gurion University of the Negev, Beer Sheva 8410501, Israel

[3]School of Chemistry, University College Cork, Cork T12 YN60, Ireland

* Corresponding authors: gedanken@mail.biu.ac.il, Fax: +972-3-7384053; Tel: +972-3-5318315

# **Equally contributed**

Inhibition/rupture of biofilm formation

The biofilm inhibition nature of the substrates was determined using two sensitive bacteria (*S. aureus* ATCC 29213, *E. coli* ATCC 25922) and two drug-resistant (MRSA ATCC 43300, MDR *E. coli* ATCCBAA 2452) laboratory strains. In all the assays, bacteria were taken in Mueller Hinton broth, grown overnight at 37°C with shaking (250 rpm), and then quantified using the absorbance (ABS595) values. The overnight cultured bacteria were diluted to 0.01 ABS for *S. aureus* and MRSA and 0.3 ABS for *E. coli* and MDR *E. coli*. For the *S. aureus* and MRSA, the experiment was performed in a 1% Mueller Hinton (MH) medium supplemented with 0.2% glucose. Each square substrate (1 cm × 1cm) substrate was placed on 24 well plates followed by adding 1 mL of the stock bacterial solution to the substrate surface. For *E. coli* and MDR *E. coli*, the experiment was carried out in 1% MH medium, and 1 mL of the bacterial stock was transferred to 24 well plates, and then the substrates were placed on it. The biofilm cultures were then allowed to grow for 18 h at 30 °C. The cultured biofilm substrates were rinsed three times with sterile doubly distilled water (DDW) to remove unattached planktonic cells. The biofilm was scrapped from the substrates using 250 µL of 1% MH medium and a cell scraper (Greiner Bio-one). The resulting solution was then transferred into a 96-well plate, and each well was filled with 180 µL of MH medium. Based on sequential dilution the bacteria were spread on LB agar plates and incubated at 37 °C for 18 h. The eradication of the bacterial biofilm was detected by the viable count method.

Sulfuric acid hydrolysis

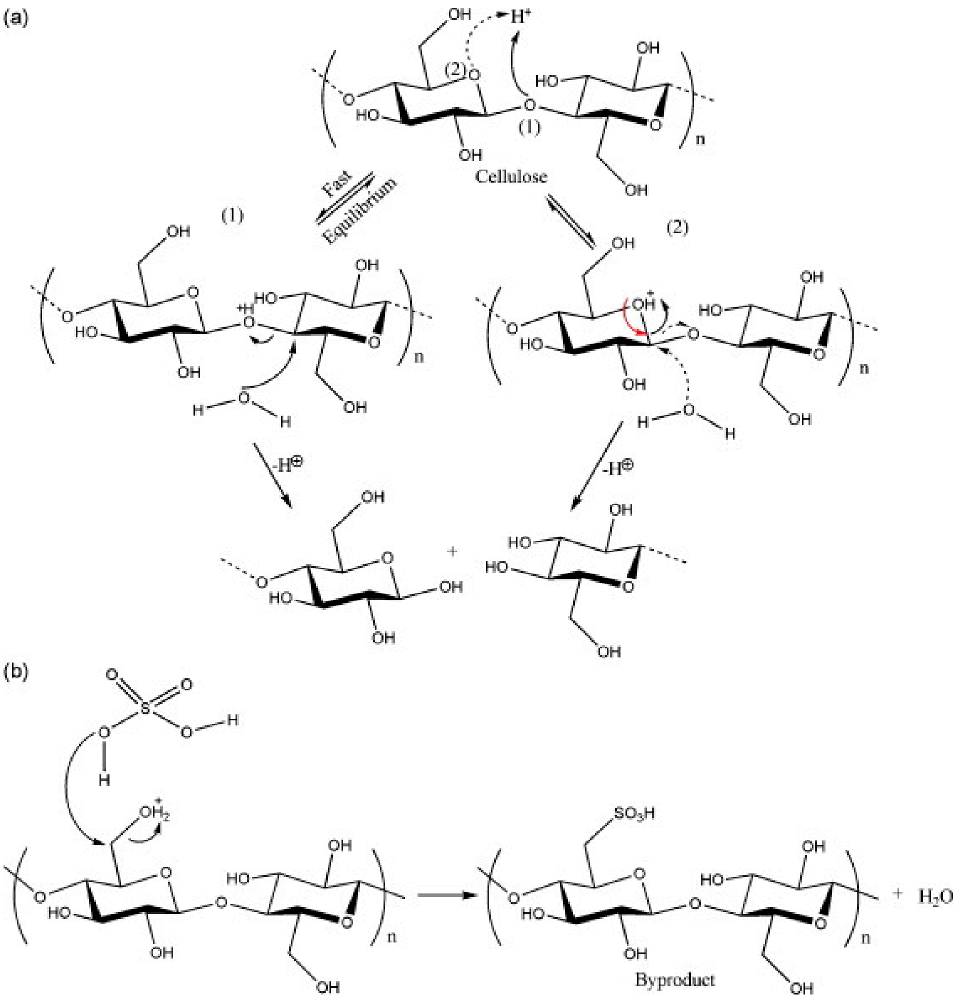

Scheme S1. (a) Acid hydrolysis mechanism. (b) Esterification of CNC surfaces

Sulfuric acid is the most used acid among the acid hydrolysis reagents for nanocrystalline cellulose production due to the fast removal of amorphous regions and the formation of stable dispersion with negative charges on the surface.

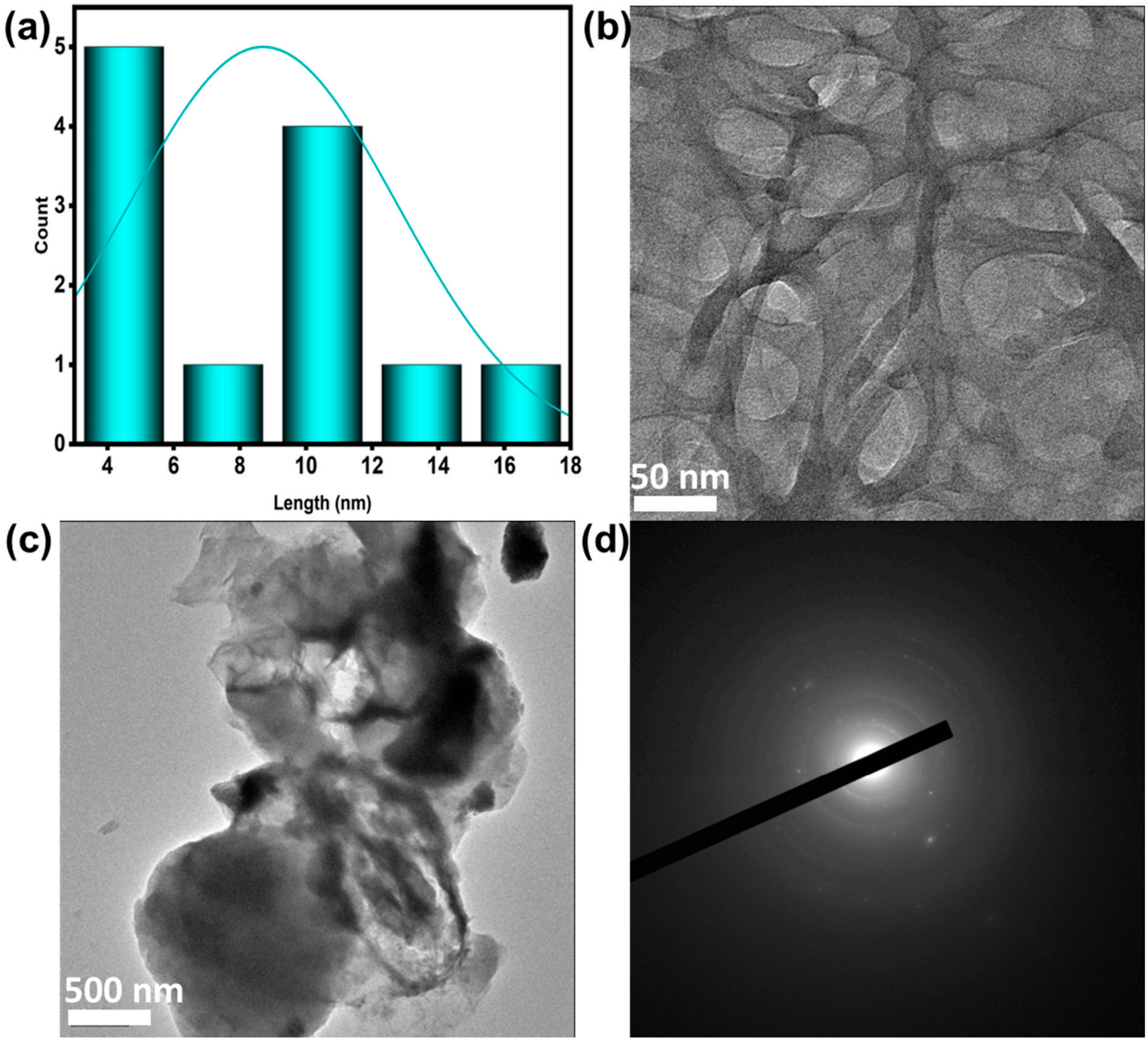

Figure S1. (a) Particle distribution analysis of commercial cellulose nanocrystals CNCs (b) TEM image of commerical CNCs (c) TEM image of commerical MCC (d) SAED pattren of MCC.
